# Cadmium Induces Apoptosis in Freshwater Crab *Sinopotamon henanense* through Activating Calcium Signal Transduction Pathway

**DOI:** 10.1371/journal.pone.0144392

**Published:** 2015-12-29

**Authors:** Jinxiang Wang, Pingping Zhang, Na Liu, Qian Wang, Jixian Luo, Lan Wang

**Affiliations:** 1 School of Life Science, Shanxi University, Taiyuan, 030006, China; 2 School of Life Science, Shanxi Datong University, Datong, 037009, China; Karolinska Institutet, SWEDEN

## Abstract

Calcium ion (Ca^2+^) is one of the key intracellular signals, which is implicated in the regulation of cell functions such as impregnation, cell proliferation, differentiation and death. Cadmium (Cd) is a toxic environmental pollutant that can disturb cell functions and even lead to cell death. Recently, we have found that Cd induced apoptosis in gill cells of the freshwater crab *Sinopotamon henanense* via caspase activation. In the present study, we further investigated the role of calcium signaling in the Cd-induced apoptosis in the animals. Our data showed that Cd triggered gill cell apoptosis which is evidenced by apoptotic DNA fragmentation, activations of caspases-3, -8 and -9 and the presence of apoptotic morphological features. Moreover, Cd elevated the intracellular concentration of Ca^2+^, the protein concentration of calmodulin (CaM) and the activity of Ca^2+^-ATPase in the gill cells of the crabs. Pretreatment of the animals with ethylene glycol-bis-(b-aminoethyl ether)-N,N,N’,N’-tetraacetic acid (EGTA), Ca^2+^ chelator, inhibited Cd-induced activation of caspases-3, -8 and -9 as well as blocked the Cd-triggered apoptotic DNA fragmentation. The apoptotic morphological features were no longer observed in gill cells pretreated with the Ca^2+^ signaling inhibitors before Cd treatment. Our results indicate that Cd evokes gill cell apoptosis through activating Ca^2+^-CaM signaling transduction pathway.

## Introduction

Cadmium (Cd) is a non-essential toxic heavy metal widely spreaded in terrestrial and aquatic environments during mining and manufacturing. Cd accumulates in organisms by feeding and metabolic processes, whose toxicity is exemplified by an extremely long biological half-life within organisms (15–30 years), resulting in both acute and chronic toxicity. Acute exposure of animals to high dose of Cd can cause cell apoptosis within a short time [1,2], while longer term exposure to small amounts of Cd can result in tissue and organ damage and cell necrosis [3, 4]. During necrosis, cells first swell and then plasma membrane collapses and cells are rapidly lysed [5]. In the process of apoptosis, its membrane is intact [6]. Apoptosis is associated with morphological changes in the cell including cell shrinkage [7], deformation in the nucleus [8], chromatin condensation [9,10], DNA fragmentation [11] and formation of apoptotic bodies [12]. Typical apoptotic features also include caspase activation [13,14]. Apoptosis is a mechanism to protect the animal from disease by removing genetically damaged cells [15].

Under Cd stress, multiple cell signal transduction pathways may be activated that control cell proliferation or death [16]. Calcium ion (Ca^2+^) is a intracellular second messenger and its overload or disturbance in its intracellular compartmentalization can trigger apoptosis in various cells types [17–21]. The increase of intracellular Ca^2+^ concentration ([Ca^2+^]i) can be elicited through two pathways: i) the Ca^2+^ release from intracellular stores, mainly the endoplasmic reticulum (ER) and Golgi apparatus, or ii) the entry from the extracellular milieu [22]. Misra et al. [23] reported that Cd may interact with cell surface membrane proteins coupled to a G protein, which drives IP_3_ induction and Ca^2+^ release from ER and Golgi in primary murine macrophages. Intracellular Ca^2+^ increase involves the opening of the plasma membrane Ca^2+^ channels. Yeh et al. [24] found that Cd induced a [Ca^2+^]i increase in Madin Darby canine kidney cells via evoking Ca^2+^ entry through non-selective Ca^2+^ channels. Cd exposure led to the increase of [Ca^2+^]i. Extracellular Ca^2+^ removal by EGTA also diminished Cd-induced [Ca^2+^]i overloading but showed slight elevation of [Ca^2+^]i, suggesting that Cd-induced extracellular Ca^2+^ influx is an important source for elevated [Ca^2+^]i, but that another source of intracellular Ca^2+^ storage is also important [25,26]. Elevated intracellular Ca^2+^ may lead to excessive Ca^2+^ uptake by mitochondria [27]. Ca^2+^ in the mitochondrial matrix interacts with cyclophilin D to induce opening of the PTP, giving rise to release of the cytochrome *c* (Cyt *c*) [28]. Cytosolic Cyt *c* binds on Apaf-1 and dATP, resulting in recruitment and activation of pro-caspase-9. Activated caspase-9 proteolytically activates caspase-3, so as to orchestrate the biochemical execution of apoptosis [29]. Hepatocyte exposure to Cd triggers the release of cytochrome *c* in the crytosol and significant caspase-3, -8 and -9 activation in rainbow trout [30]. Calmodulin (CaM) is a ubiquitous eukaryotic Ca^2+^-dependent protein. Its activity is closely associated with the intracellular concentration of Ca^2+^. Binding of Ca^2+^ to CaM activates CaM through configurational changes, which impacts the structure and activity of more than 20 enzymes [31]. Chen et al. [7] reported that Ca^2+^/CaM-dependent protein kinase Ⅱ is activated by Cd triggering neuronal cell apoptosis. [Ca^2+^]i is usually regulated by Ca^2+^-ATPase, which can be activated by a small increase in [Ca^2+^] and export Ca^2+^ from the cytosol to the extracellular environment. Studies have shown that Cd disrupts intracellular Ca^2+^ homeostasis, leading to apoptosis in various types of cells [26,32,33].

The freshwater crab *Sinopotamon henanense* is a benthonic macroinvertebrate which inhabits aquatic environments and can accumulate metals *in vivo* [1,34]. In the previous studies, we found that the 96 h LC_50_ value of Cd to *S*. *henanense* is 232 mg•L^-1^ for adult male crabs [35,36]. Acute exposure to high concentrations of Cd resulted in cell apoptosis in the gill, hepatopancreas and testes of *S*. *henanense* [10,37,38]. Cd also led to Ca^2+^-ATPase activation in hepatopancreas of the animal. There have been no specific studies in freshwater crab to measure cytosolic Ca^2+^ concentration after exposure to waterborne Cd. And the effect of Cd on intracellular Ca^2+^ signal and the molecular regulation mechanism of Ca^2+^ on Cd-induced apoptosis are poorly understood. In the present study, we investigated the role of Ca^2+^ signaling in the Cd-evoked apoptosis in gills of the *S*. *henanense*.

## Materials and Methods

### Chemicals and reagents

All the chemicals used were obtained from Sigma Co. (St. Louis, MO, USA). Assay kits for caspase-3, caspase-8 and caspase-9, the kit for DNA purification and Fluo-3/AM were obtained from Beyotime Institute of Biotechnology (Jiangsu Province, China). CaM and Ca^2+^-ATPase analysis kits were supplied by Jiancheng Bioengineering Institute (Jiangsu Province, Nanjing, China).

### Animals and exposures

Freshwater crabs, *S*. *henanense*, were purchased from the Dongan aquatic market in Taiyuan city, PR China. Prior to the experiments, crabs were acclimated for 2 weeks in glass aquaria filled with dechlorinated, carbon-filtered city tap water (pH 7.5, dissolved oxygen 8.0–8.3 mg/L) at a temperature of 20 ± 2°C and a photoperiod of 12/12 h (day / night) and were fed with commercial food three times a week. Only healthy adult male crabs weighted 20.0 ± 0.5 g were employed in this study. The crabs were divided into four experimental groups of twenty specimens each and allocated to control and three Cd^2+^ concentrations of at 14.5, 29 and 58 mg•L^-1^ (corresponding to 1/16, 1/8, 1/4 of the 96 h-LC_50_) for 96 h at 22°C in glass aquaria [36]. To study the effects of inhibitor, the crabs were pretreated with 5 mM EGTA for 4 h, then exposed to 58 mg•L^-1^ CdCl_2_ for 48 h under the same conditions as described above. The exposure medium was changed every 48 h. During the experiment, crabs were not fed until the end of the exposure, the gill of crab was removed and immediately frozen in liquid N_2_ for use.

### Measurement of [Ca^2+^]

To measure [Ca^2+^], the gill cells were isolated from the untreated crab gill and were loaded with 5 μM Fluo-3/AM for 30 min at 37°C in the dark. After incubation, the cell suspensions were washed twice with PBS to remove the extracellular Fluo-3/AM. Then the cells were treated with 5.8 mg•L^-1^ CdCl_2_ for the different times. Fluorescent probe was excited at 488-nm, and emission fluorescent was filtered by a 525-nm filter. The images were recorded under a laser confocal microscope (LSM 510 META, Leica, Bensheim, Germany).

### Determination of CaM protein concentration

CaM content assays were performed using the methods as described by Liu et al. [39] and Hu et al. [40] with slight modifications. To isolate CaM, gill tissue was homogenized in buffer solution (50 mM Tris-HCl, pH 8.0, 1 mM EGTA, 0.5 mM phenylmethylsulfonyl fluoride) on ice. The homogenate was treated in a water bath at 90°C to 95°C for 3 min and cooled at 4°C, and then centrifuged at 10,000 × g for 30 min. The supernatant was used for measurement of total protein concentration and CaM concentration. Total protein concentration was determined according to the method of Bradford [41] with bovine serum albumin (BSA) as standard. The CaM concentration was determined by enzyme-linked immunosorbent assay (ELISA) according to the protocol recommended by the manufacturer.

### Measurement of Ca^2+^-ATPase activities

Activities of Ca^2+^-ATPase were determined spectrophotometrically by using a kit from the Nanjing Jiancheng Bioengineering Institute according to the protocol recommended by the manufacturer.

### DNA-fragmentation assay

DNA was extracted with the DNA purification kit (Beyotime, C0008) and DNA fragmentation pattern was assessed by conventional agarose gel electrophoresis. Briefly, at the indicated time points after treatments, 20 mg of the sample was collected. The eluates containing DNA were loaded for electrophoresis on a 1.5% agarose gel at 100 V for 1 h. Gels were stained with ethidium bromide. Pictures of DNA bands were visualized and captured by ultraviolet gel documentation system.

### Ultramicrostructure observation using transmission electron microscopy

After Cd exposure, three to five tissue pieces from the middle of gill lamellae (approximately 1 to 2 mm^2^) were cut and then stored in glutaraldehyde. After fixation, tissues were rinsed twice in the buffer immediately, post-fixed in 1% osmium tetroxide, dehydrated in a graded ethanol series and embedded in thin viscosity resin. Ultrathin sections were cut with an ultramicrotome (Leica UC-6), stained with uranyl acetate and lead citrate, and examined using a transmission electron microscope (JEM-1011) at an accelerating voltage of 80 kV.

### Caspase activity assay

Assays for caspase-3, -8 and -9 activities were performed using Caspase Activity Assay kits (Beyotime, C1115, C1151, and C1157). Approximately 30 mg of frozen gill segments were homogenized in 300 μl of lysis buffer and centrifuged at 15,000 g for 20 min (4°C). The supernatant was immediately used for caspase activity assays. Caspase activity was measured through cleavage of colorless substrates Ac-DEVD-pNA, Ac-IETD-pNA and Ac-LEHD-pNA for caspase-3, caspase-8 and caspase-9 respectively. An increase in absorbance at 405 nm was used to quantify enzyme activity. Caspases-3, 8 and 9 activities were calculated using a standard curve. One unit is the amount of enzyme that can produce 1.0 nmol of the colorimetric substrate pNA per hour at 37°C under saturated substrate concentrations.

### Statistical analysis

Data were expressed as mean ± SE. Statistical analyses were performed using SPSS 17.0 computer software package. Differences among groups were examined by one-way analysis of variance (ANOVA). The post hoc least significant difference (LSD) test was performed for inter-group comparisons. Values of *p* < 0.05 were considered to be statistically significant.

## Results

### Cadmium induced apoptosis in gill cells

**Fig 1:**
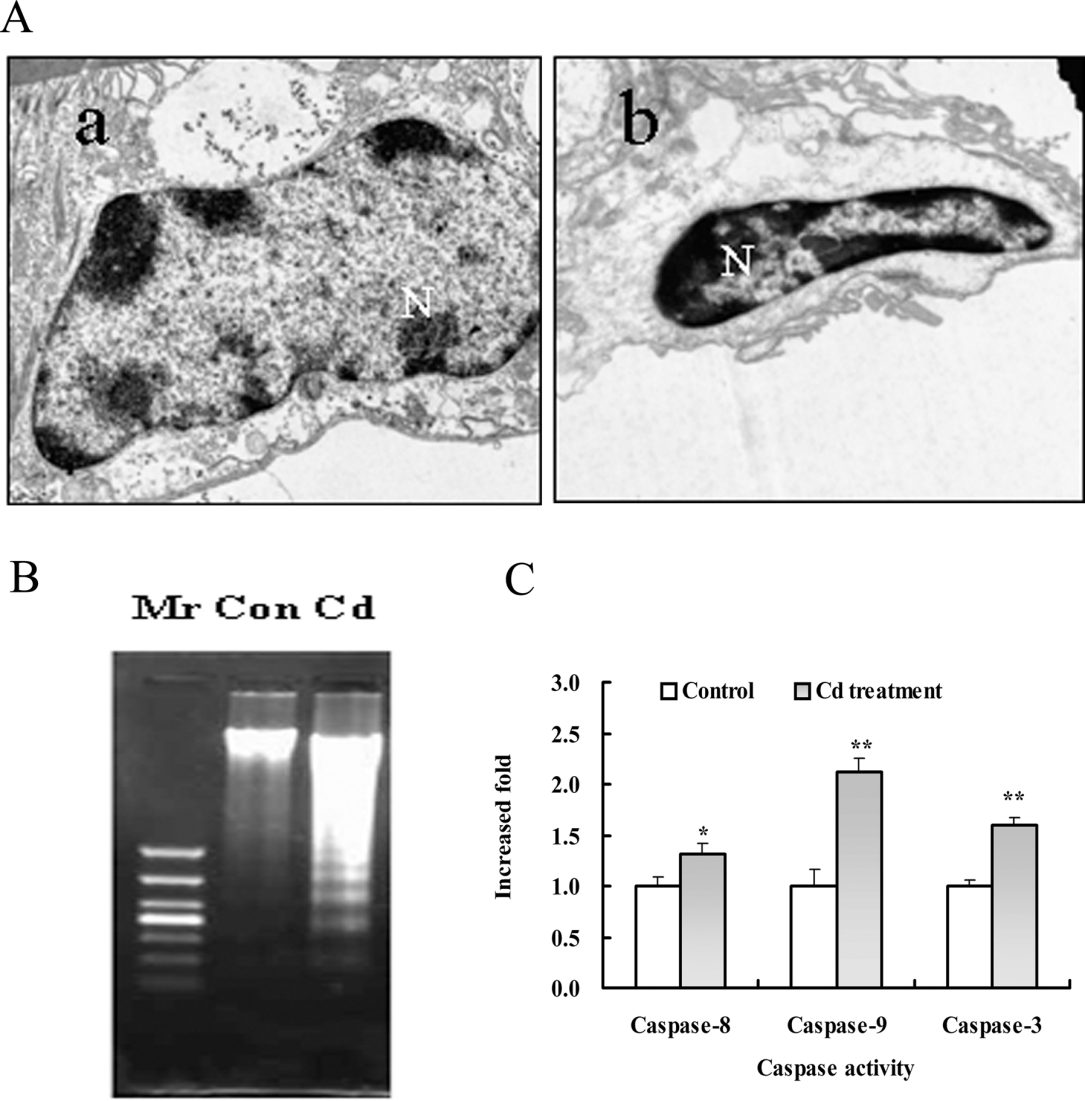
Cd induced apoptosis in gill cells of freshwater crab *S*. *henanense*. Crabs were treated with 58 mg•L^-1^ CdCl_2_ for 48 h and cell apoptosis was assessed. (A) The effects of Cd on the morphology of nuclei. (a) (×8000) normal nuclei in untreated control group. (b) (×8000) abnormal nuclei with apoptotic characteristics in the Cd-treated group. (B) DNA fragmentation characteristics of gill cells by Cd. Mr: DNA marker, Con: control (untreated gill cells), Cd: Cd-treated gill cells. (C) The effects of Cd on the activities of caspases-3, -8 and -9. The mean expression in each treated group is shown as a fold increase compared to the mean expression in the control, which had been ascribed an arbitrary value of 1. *P<0.05, **P<0.01 difference vs. control group.

In order to investigate the cytotoxicity of Cd and effects of Cd on apoptosis in the gill epithelial cells, crabs were exposed to 58 mg•L^-1^ CdCl_2_ for 48 h. There are a large number of organelles in the control epithelial cell (Fig 1Aa). Organelles were reduced, and the phenomenon of vacuoles was obvious in the Cd-treated cell (Fig 1Ab), indicating that cell damage were serious after 48 h of exposure to Cd. Ultramicrostructure of the epithelial cells showed that the control nuclei were normal and heterochromatin was uniformly distributed and adherent to the continuous and intact nuclear envelope (Fig 1Aa). Contrast with the control group, Cd led to the shrinkage of nucleus and chromatin condensation (Fig 1Ab). DNA fragmentation ladder was found in the Cd-treated gill cells (Fig 1B). Fig 1C showed that caspase-3, -8 and -9 were activated in the gill cells from the exposed crabs to Cd for 48 h. These results indicated that Cd is able to induce apoptosis in gill, which was consistent with our previous findings [10].

### Cadmium increased the level of intracellular [Ca^2+^]

**Fig 2:**
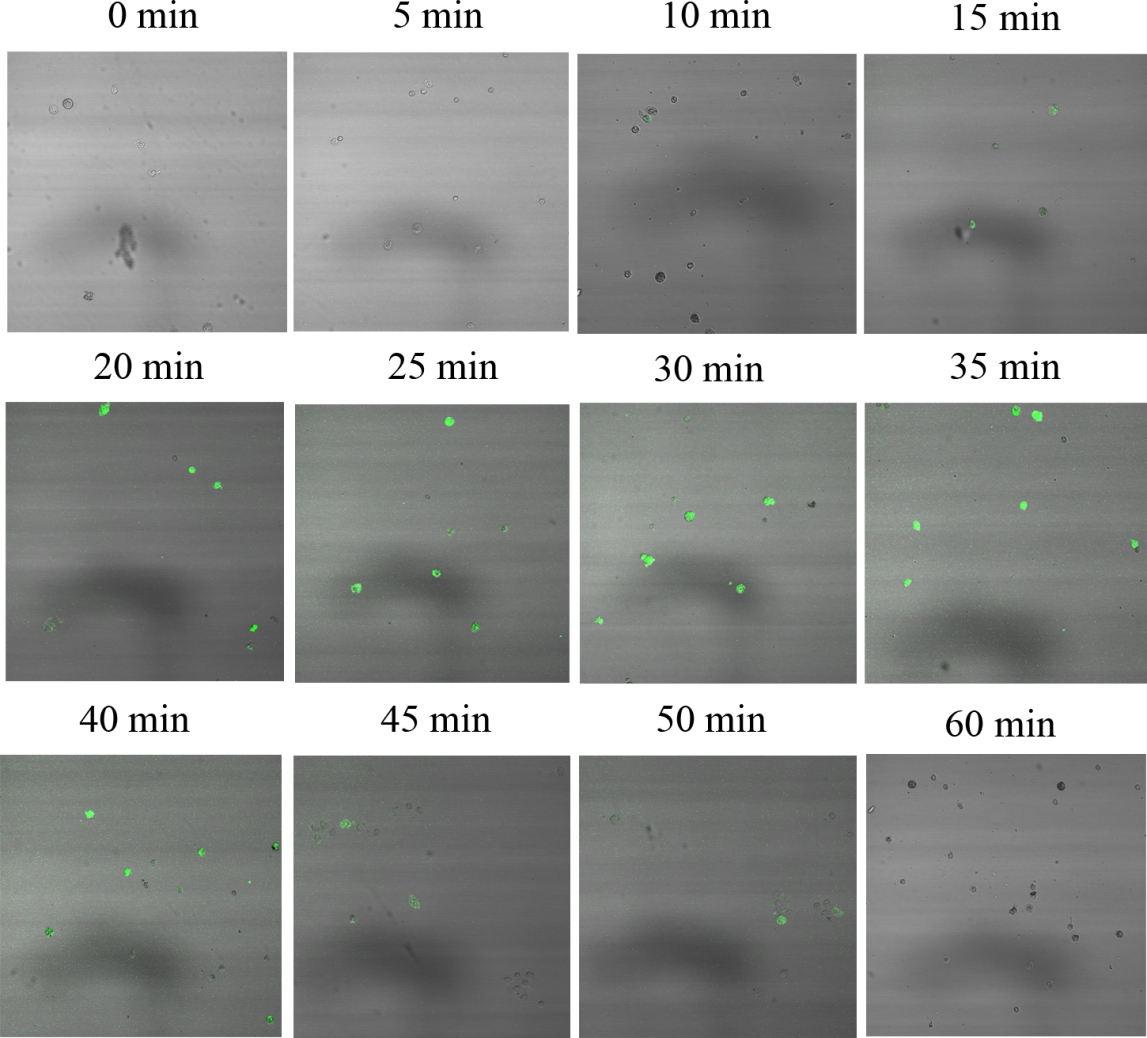
Effects of acute Cd exposure on [Ca^2+^]i in gill cells of *S*. *henanense*. Cells were loaded with 5 μM Fluo-3/AM for 30 min at 37°C in the dark, then treated with 5.8 mg•L^-1^ CdCl_2_ for the different times, followed by the images recording under a laser confocal microscope (LSM 510 META, Leica, Bensheim, Germany).

To investigate whether the Cd-induced apoptosis is associated with [Ca^2+^], we first determined the [Ca^2+^] level using the Ca^2+^ indicator Fluo-3/AM. Cells from untreated crab gill were loaded with Fluo-3/AM, and then treated with 5.8 mg•L^-1^ CdCl_2_, and observed by laser confocal microscope (LSCM). A time-course study on intracellular [Ca^2+^] level alteration was performed with Cd treatment for 0 to 60 min. As shown in Fig 2, compared with the baseline level of [Ca^2+^] in control cells, intracellular [Ca^2+^] level in Cd-treated cells for 20 min started to increase significantly and reached its peak at 35 min. The [Ca^2+^]i at 60 min was similar to that of control cells.

### The change in CaM content by Cadmium-induction

**Fig 3:**
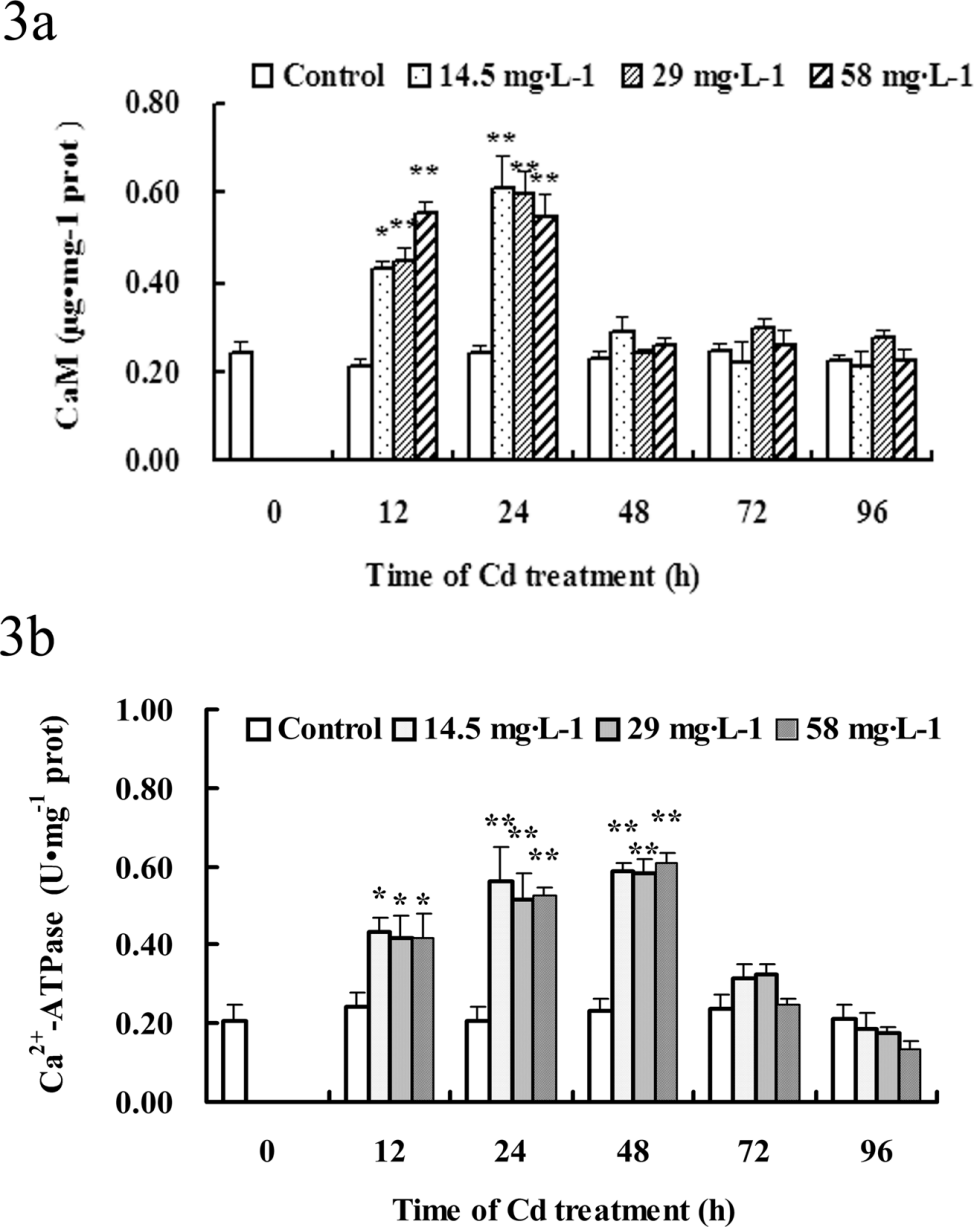
Effects of Cd exposure on CaM content and Ca^2+^-ATPase activity in gill cells. Results are presented as mean ± SE; n = 3. *P<0.05, **P<0.01 difference vs. control group.

Cd treatment led to the changes of [Ca^2+^]i in gill cells of *S*.*henanense*. To investigate the effects of Cd on CaM, the CaM content in the Cd signaling were measured using ELISA method. As shown in Fig 3A, in the control group, there were no significant changes in the content of CaM in gill cells. However, Cd treatment caused clear increase in the content of CaM. The content of CaM significantly increased within 12 h of Cd treatment, reached a maximum at 24 h, and then the increase trend started to drop.

### Cadmium increased the Ca^2+^-ATPase activity

As shown in Fig 3B, the activity of Ca^2+^-ATPase was determined over an experimental period of 96 h. During this period, the activity of the control varied only slightly around their means. In the presence of Cd the activity of Ca^2+^-ATPase was obviously increased within 12 h. After 48 h of treatment with Cd, Ca^2+^-ATPase activity reached maximum values, being 2-, 2.1- and 2.1- folds, respectively, higher than those in the controls for 14.5, 29 and 58 mg•L^-1^ Cd treatment group. And the activity of Ca^2+^-ATPase decrease after 72 h of Cd treatment.

### Effects of Ca^2+^ inhibitor on Cadmium-induced DNA fragmentation

**Fig 4:**
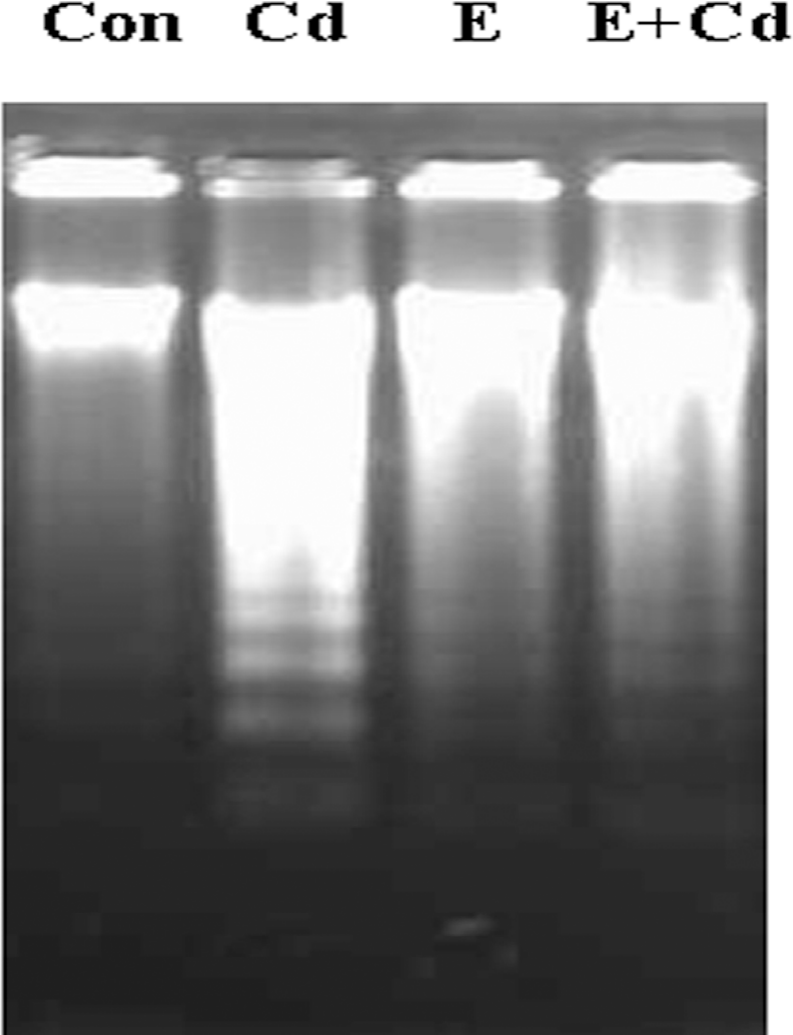
Effects of pretreatment with EGTA on DNA fragmentation induced by Cd. DNA fragmentation was assayed in agarose gel electrophoresis. Crabs were pretreated with or without 5 mM EGTA for 4 h, and then exposed to 58 mg•L^-1^ Cd for 48 h. The letters on the lanes represent: Con = H_2_O (4 h) + H_2_O (48 h); Cd = H_2_O (4 h) + 58 mg•L^-1^ CdCl_2_ (48 h); E = 5 mM EGTA (4 h) + H_2_O (48 h); and, E + Cd = 5 mM EGTA (4 h) + 58 mg•L^-1^ CdCl_2_ (48 h).

Cd could lead to the increase of [Ca^2+^] and induce apoptosis in gill cells. We proposed that the change of [Ca^2+^] is involved in the Cd-induced cytotoxicity. To establish a link between Ca^2+^ and apoptosis in Cd signaling, crabs were treated with Ca^2+^ inhibitor (EGTA) for 4 h prior to Cd treatment. As shown in Fig 4, acute Cd exposure resulted in DNA apoptotic fragmentation in gill cells of *S*. *henanense*. Pretreatment of Ca^2+^ inhibitor blocked the DNA fragmentation, indicating that Cd-elevated [Ca^2+^] is required for the apoptotic DNA fragmentation induced by Cd.

### Effect of pretreatment with Ca^2+^ inhibitor on ultrastructure in gill cells

**Fig 5:**
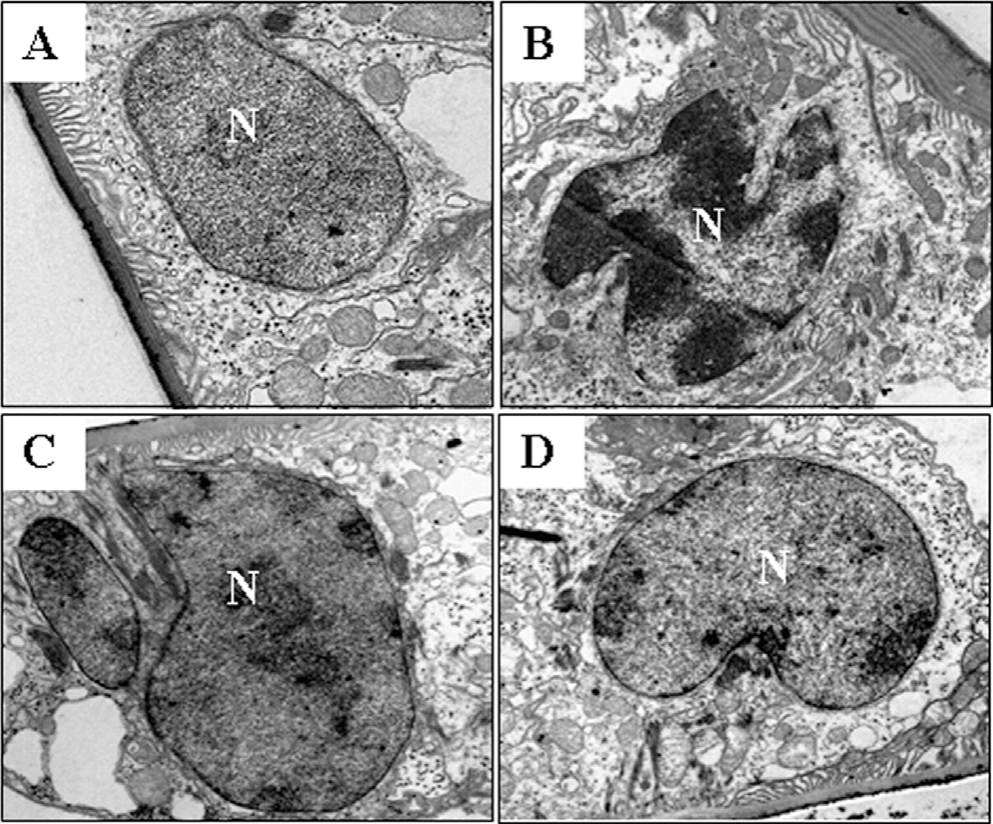
Effects of EGTA pretreatment on Cd-induced morphological variation of epithelial cells in the gill. (A) (×10000) normal epithelial cell with nucleus and a large of cytoplasmic organelles. (B) (×10000) apoptotic epithelial cell induced by 58 mg•L^-1^ CdCl_2_ with apoptotic characteristics, such as chromatin condensation and extremely irregular nuclear membrane in nucleus. (C) (×8000) epithelial cell treated by 5 mM EGTA alone; (D) (×6000) epithelial cell treated by 5 mM EGTA for 4 h, then exposed to Cd for 48 without apoptotic characteristics.

To further test the effects of Cd on apoptosis in gill cells and regulation of Ca^2+^ signaling on Cd-induced apoptosis, ultrastructure of gill cells were analyzed using transmission electron microscopy (Fig 5). These results revealed that the nuclei of control epithelial cells were normal and heterochromatin was uniformly distributed (Fig 5A). In the presence of Cd, typical apoptotic characteristics in nuclei of gill cells were observed, such as chromatin condensation and irregular nuclei with fingerlike buds (Fig 5B). In the EGTA alone treatment group, morphologies of gill cells and nuclei were normal without apoptotic features (Fig 5C). In EGTA plus Cd group (Fig 5D), the characteristics of apoptotic features were also not observed in the cells. Thus, pretreatment of Ca^2+^ inhibitor inhibited Cd-induced apoptosis of gill cells.

### Effect of pretreatment with Ca^2+^ inhibitor on caspase-3/8/9 activities

**Fig 6:**
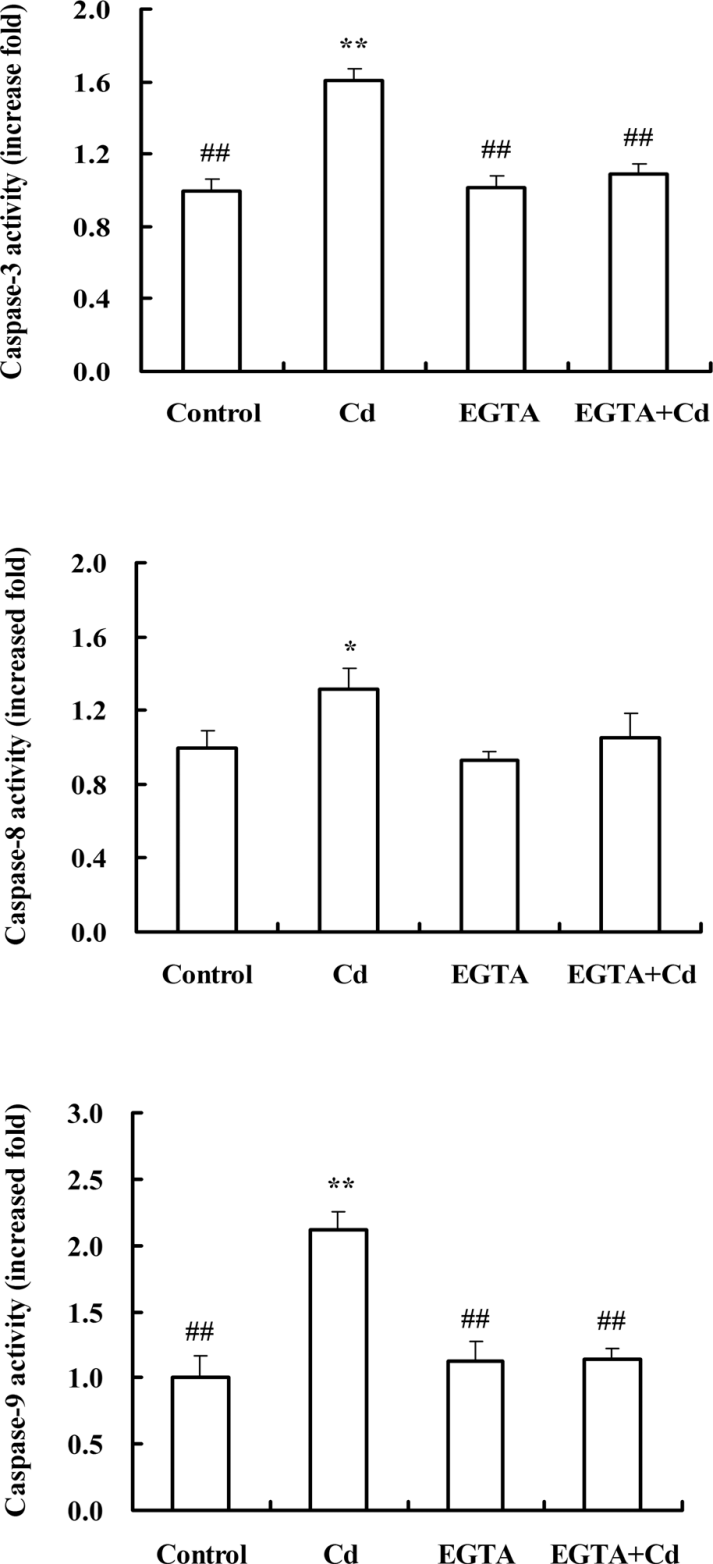
Effects of pretreatment with EGTA on the activities of caspase enzymes in the gill of crabs exposed to acute Cd treatment. The mean expression in each treated group is shown as a fold increase compared to the mean expression in the control, which had been ascribed an arbitrary value of 1. Using one-way analysis of variance and on comparing with the control, significance is shown by *P<0.05, **P<0.01; on comparing with Cd treatment group, ^#^ *P*<0.05, ^##^ *P*<0.01.

Treatment with Cd also led to significant increase in the activities of caspases-3, -8 and -9 in the gill of *S*. *henanense* compared with the control (Fig 6). Pretreatment with EGTA blocked the Cd-induced increase in the activities of these caspase enzymes (Fig 6).

## Discussion

Exposure of cells to Cd can evoke a number of cellular responses to protect the cell from the metal-induced cytotoxicity. The primary protective mechanism is by sulfhydryl compounds such as glutathione and metallothionein, which can decrease the intracellular free Cd ions by sulfhydryl reaction [1,35,42,43]. The second mechanism is the activation of DNA repair proteins such as heat shock proteins (HSP), protein disulfide isomerase (PDI) and DNA glycosylase [44–46]. Ultimately, when the damage caused by Cd ions exceeds the capacity of cell to repair such damage, the cell can activate signaling pathways to initiate apoptosis, which is believed to limit the damage locally to prevent injury spreading in organisms. Ca^2+^ is an important signal in the regulation of various cell functions such as fertilization, proliferation, development, learning and memory, contraction and secretion [17,47,48]. The [Ca^2+^]i is tightly controlled in all eukaryotic cells. Typically, [Ca^2+^] in the cytosol is within the range 0.05–0.5 μM and is 1–10μM in the extracellular fluid [49]. On chemical stimulation, the intracellular [Ca^2+^] increases by one to two orders of magnitude either as a result of Ca^2+^ influx across the cell membrane, or through the release of Ca^2+^ from intracellular stores, or both [50]. In the mesangial cells, Cd treatment led to an increase in [Ca^2+^]i, which resulted from the release of Ca^2+^ from ER in 0.5 to 8.5 h [51]. Similar results were obtained in macrophages exposed to Cd, although the increase of [Ca^2+^]i was detected in shorter time range (300 s) [23]. Chen et al. [7] and Xu et al. [26] have found that Cd (0–20 μM) elevated [Ca^2+^]i level in PC12 and SH-SY5Y cells as well as primary murine neurons in a concentration- and time-dependent manner, which lasted for 24 h. Liu et al. [52] reported that Cd caused the rapid elevation of [Ca^2+^]i, which occurred as early as 0.5 h following treatment with Cd (100 μM), and cell apoptosis in thyroid cancer cells. In the present study, we found that Cd exposure altered the balance of intracellular Ca^2+^ and resulted in the increase of [Ca^2+^]i, which is consistent with the observations by Kim and Sharma [25] who demonstrated that low concentration Cd (i.e., 20 μM) caused a low-amplitude [Ca^2+^]i elevation and high concentration Cd (i.e., 500μM) resulted in rapid and high-amplitude [Ca^2+^]i elevation. We treated the gill cells of crab with 5.8 mg•L^-1^ Cd (i.e., 51.6 μM) and found that [Ca^2+^] started to increase within 20 min and the increase started to drop after 40 min (Fig 2).

Many proteins cannot bind calcium themselves and have to use intracellular Ca^2+^-binding proteins as a calcium sensor and signal transducer to modulate cellular processes [26]. CaM is a highly conserved Ca^2+^-binding protein in eukaryotic cells containing the EF-hand structural motif, which functions as cofactors in different Ca^2+^-dependent processes. CaM itself has no catalytic activity but, upon Ca^2+^ binding, it activates numerous target proteins involved in a variety of cellular processes including apoptosis [53,54]. These Ca^2+^/CaM stimulated proteins include various Ser/The protein kinases, protein phosphatase calcineurin, nitric oxide synthases, ion transporters and cytoskeletal proteins. Cd is reported that may induce [Ca^2+^]i elevation, and Cd-elevated [Ca^2+^]i activates MAPK and CaMKII, which are activated in the presence of Ca^2+^ and CaM [7]. The NFAT (nuclear factor of activated T cells) proteins are at the heart of Ca^2+^ signal transduction in cells [55]. Ca^2+^ binds to calcineurin as well as to CaM to activate the phosphatase activity of calcineurin. Calcineurin dephosphorylates multiple phosphoserines on NFAT, leading to its nuclear translocation and activation. In most cases, NFAT-dependent transcription requires that the Ca^2+^ signaling be coincident with MAP kinase signaling [56]. NFAT have been shown to play roles in apoptosis regulation both in immune and nonimmune cells [57]. Álvarez et al. [58] reported that nuclear translocation of NFAT through activation of calcineurin after TNF-a treatment leads to increased apoptosis in neuroblastoma cells. Many, and possibly most, Ca^2+^-induced genes are regulated by the transcription factor CREB (cyclic AMP response element—binding protein) [59]. CREB appears to be a primary transcriptional activator of the anti-apoptotic gene, bcl-2 [60]. Inhibition of CREB activity induces apoptosis. CREB protein is a target for caspases, which is cleaved by caspases during neural cell apoptosis [61]. The destruction of CREB eliminates a key factor that could reverse apoptosis. CaMK4 regulates cell apoptosis and proliferation in part via CREB activation in β-cells [62]. Many studies have shown that CaM is activated by metal ions [63,64]. Silencing CaM remarkably inhibited Cd-induced phosphorylation of MAPK and Akt/mTOR pathways and cell death in PC12 cells [26]. We investigated the effect of Cd on CaM content in the present study. The data showed that Cd increased the CaM content of gill in *S*. *henanense*, indicating that Ca^2+^ and CaM are regulated by Cd (Fig 3A).

The recovery of basal [Ca^2+^] after treatments is carried out by energy-consuming Ca^2+^-ATPases and Na^+^/Ca^2+^-exchange mechanisms [65]. Ca^2+^-ATPase is the one situated in the plasma membrane and intracellular membranes of erythrocytes, which had high affinity for Ca^2+^ [66]. The Ca^2+^-activated ATPase is normally considered to be a "defense system" that re-establish basal [Ca^2+^] after a Ca^2+^ signaling event. Ca^2+^-pumps might also contribute to the generation/modulation of Ca^2+^ signals [67]. In this study we found that Cd elicited a transit increase in cytosolic [Ca^2+^] (Fig 2), along with which was the increase of Ca^2+^-ATPase activity (Fig 3B). This indicates that the increased enzymatic activity may be used to pump out excessive Ca^2+^ in the cytosol [37]. In the later stages of Cd treatment, Ca^2+^-ATPase activity had no significant change compared with the control in crabs (Fig 3B), indicating that [Ca^2+^] is not activated at the late time.

Cd can mediate a wide variety of cytotoxic and metabolic effects, such as altering the activities of various enzymes, interfering with the normal protective actions of essential metals, inducing oxidative stress, inhibiting mitochondrial ATP production, and altering gene expression that may trigger cell death by either apoptosis or necrosis [68]. Cd led to cells apoptosis in the gill, hepatopancreas and testes of *S*. *henanense* [1,37,38] and changed the Ca^2+^-ATPase activity [37,69]. H_2_O_2_ production is involved in the Cd-induced cell apoptosis [10]. However, the role of Ca^2+^ signal in the apoptosis pathway is still unknown. In the present study, we found that Cd induced a rapid and transient, but significant, cytosolic [Ca^2+^] elevation, followed by apoptosis in gill cells of the crabs. The Ca^2+^ inhibitor, EGTA, could completely antagonize the apoptotic action of the metal, through the inhibition of activation of caspases-3, -8 and -9 and preventing DNA fragmentation. The result demonstrates that removal of Ca^2+^ from medium (by chelator EGTA) protects cells from apoptosis induced by Cd. This finding is in keeping with the results by other researchers [7,25,26,70], which demonstrate that Cd can induce [Ca^2+^] elevation leading to cell apoptosis in J774A.1 (murine macrophages), PC12 (rat pheochromocytoma) and SH-SY5Y (human neuroblastoma) cell lines, and mouse thymocytes. Calcium ions are the most powerful inductors and mediators of cell death. Ca^2+^ signal is involved in the regulation of cell apoptosis induced by Cd in *S*. *henanense*. However, Ca^2+^ cross-talks with a variety of signaling pathways and the integrative function of the Ca^2+^ signaling in apoptosis requires further investigations.

## Conclusion

Our results showed that acute Cd exposure increased [Ca^2+^]i level and Ca^2+^-ATPase activity as well as the protein concentration of CaM, which is followed by gill cell apoptosis which was evidenced by activation of caspases- 3, -8 and -9, apoptotic DNA fragmentation, and apoptotic ultrastructure morphology. All the apoptotic features in the gill cells induced by Cd were inhibited by pretreatment of the animals with Ca^2+^ chelator EGTA. Thus, it is indicated that Cd elicits gill cell apoptosis through Ca^2+^ signal transduction pathway in the animal model.
